# The role of endobronchial ultrasonography elastography in the diagnosis of hilar and mediastinal lymph nodes

**DOI:** 10.55730/1300-0144.5634

**Published:** 2023-02-07

**Authors:** Barış DEMİRKOL, Elif TANRIVERDİ, Şule GÜL, Aysu Sinem KOÇ, Elife AKGÜN, Aytül Hande YARDIMCI, Kürşad Nuri BAYDİLİ, Erdoğan ÇETİNKAYA

**Affiliations:** 1 Department of Pulmonology, Başaksehir Çam and Sakura City Hospital, University of Health Sciences, İstanbul, Turkey; 2 Department of Pulmonology, Yedikule Chest Diseases and Thoracic Surgery Education and Research Hospital,University of Health Sciences, İstanbul, Turkey; 3 Department of Pulmonology, Bahçeşehir Liv Hospital, İstinye University, İstanbul, Turkey; 4 Department of Nuclear Medicine, Kırıkkale Yüksek İhtisas Hospital, Kırıkkale, Turkey; 5 Department of Radiology, Başakşehir Çam and Sakura City Hospital, University of Health Sciences, İstanbul, Turkey; 6 Department of Biostatistics and Medical Informatics, Faculty of Hamidiye Medical, University of Health Sciences, İstanbul, Turkey

**Keywords:** Elastography, endobronchial ultrasonography, lymph node, mediastinum, strain ratio

## Abstract

**Background/aim:**

Endobronchial ultrasonography (EBUS) is a minimally invasive diagnostic tool in the diagnosis of mediastinal lymph nodes (LNs) and has sonographic features. We aimed to investigate the diagnostic accuracy of EBUS elastography, which evaluates tissue compressibility integrated into EBUS, on malignant vs. benign mediastinal-hilar LNs.

**Materials and methods:**

A single-center, prospective study was conducted at the University of Health Sciences Yedikule Chest Diseases and Thoracic Surgery Training and Research Hospital between 01/10/2019 and 15/11/2019. The features of 219 LNs evaluated by thoracic computed tomography (CT), positron emission tomography (PET)/CT, EBUS sonography and EBUS elastography were recorded. The LNs sampled by EBUS-guided fine needle aspiration were classified according to EBUS elastography color distribution findings as follows: type 1, predominantly nonblue (green, yellow, and red); type 2, part blue, part nonblue; type 3, predominantly blue. The strain ratio (SR) was calculated based on normal tissue with the relevant region.

**Results:**

The average age of 131 patients included in the study was 55.86 ± 13 years, 76 (58%) were male. Two hundred and nineteen lymph nodes were sampled from different stations. Pathological diagnosis of 75 (34.2%) LNs was malignant, the rest was benign. When EBUS B-mode findings and pathological results were compared, sensitivity was 65.33%, specificity 63.19%, positive predictive value (PPV) 48%, negative predictive value (NPV) 77.8%, and diagnostic yield (DY) 64%. When the pathological diagnoses and EBUS elastography findings were compared, while type 1 LNs were considered to be benign and type 3 LNs malignant, sensitivity 94.12%, specificity 86.54%, PPV 82.1%, NPV 95.7%, and DY 89.5%. SR of malignant LNs was significantly higher than benign LNs (p < 0.001). When the classification according to color scale and SR were compared, no difference was found in DY (p = 0.155).

**Conclusion:**

The diagnostic accuracy of EBUS elastography is high enough to distinguish malignant LN from benign ones with the SR option. When compared with EBUS-B mode sonographic findings, it was found to have a higher diagnostic yield.

## 1. Introduction

Many benign and malignant diseases can affect the hilar and mediastinal lymph nodes. Various noninvasive and invasive methods are used in the diagnosis of mediastinal lymphadenopathies. Although positron emission tomography (PET)/ computed tomography (CT), which is one of the noninvasive methods used in the diagnosis of malignant lymph nodes has a high sensitivity, it has a low specificity due to high fluorodeoxyglucose (FDG) uptake as in granulomatous diseases such as tuberculosis, sarcoidosis, and nonspecific infections. Endobronchial ultrasonography-transbronchial needle aspiration (EBUS-TBNA) is the first preferred and most frequently used minimally invasive diagnostic method used in the diagnosis of mediastinal lymph nodes thanks to its high sensitivity, specificity, and safety. Prediction of the malignant lymph nodes detected during the EBUS procedure will provide a shorter duration of the procedure and increase the patient’s tolerance to the procedure. Therefore, EBUS-B mode sonographic findings such as the size, border features, shape, echogenicity of the lymph node, and presence of fatty hilar and necrotic region used were evaluated and found to be useful. In various studies, the diagnostic success rate in the evaluation of lymph nodes of the EBUS-B-mode ranges between 40% and 80% [1,2,3]. There are also studies showing that the vascular pattern observed in the Doppler mode during EBUS predicts nodal metastasis in patients with lung cancer [4].

Elastography is a brand-new ultrasonographic technique and the frequency of its use in different branches of the clinic keeps increasing [5]. Elastography enables healthcare professionals to make evaluations based on tissue compressibility or tissue stiffness. It has been shown in the studies that ultrasonography (US)/endoscopic ultrasonography (EUS) elastography, which was first tried in breast and pancreatic lesions, has high sensitivity and specificity in the detection of lymph nodes, liver, and thyroid malignancies [6–11]. In studies performed by integrating elastography into EBUS, it has been reported that EBUS elastography may be useful for the evaluation of mediastinal lymph nodes [5,12]. In our study, we aimed to investigate the diagnostic accuracy of EBUS elastography in the differentiation of malignant and benign lymph nodes.

## 2. Materials and methods

A single-center, prospective study was conducted in the Broncology Unit at the University of Health Sciences Yedikule Chest Diseases and Thoracic Surgery Training and Research Hospital between 01/10/2019 and 15/11/2019. One hundred and thirty-one patients with mediastinal and hilar lymph nodes detected in thorax CT and/or PET/CT examinations and EBUS indication were included in the study.

### 2.1. Inclusion criteria of the study

The inclusion criteria of the study were as follows:

- Female and male adults >18 years old,
- Patients presenting to our clinic with hilar and/or mediastinal lymphadenopathy >10 mm on thorax CT and/or activity involvement in PET/CT,
- Patients with lymph node <10 mm on thorax CT but with hilar and/or mediastinal lymphadenopathy showing activity involvement on PET/CT,
- Patients diagnosed with malignancy with hilar and/or mediastinal lymphadenopathy 10 mm in thorax CT and/or activity involvement on PET/CT for staging purposes.

### 2.2. Exclusion criteria of the study

The exclusion criteria of the study were as follows:

- The patient or his/her relatives’ refusal to undergo the procedure,
- Unsuitability of mediastinal lymph node size and localization for EBUS procedure,
- Patients in whom bronchoscopy is contraindicated.

**Figure 1:**
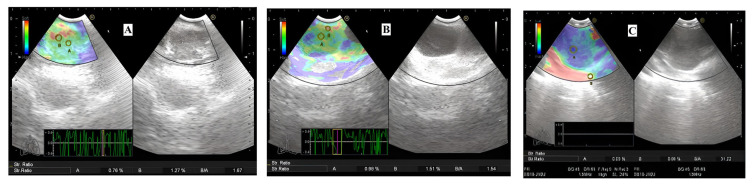
Classification of the lymph nodes according to color distribution with EBUS elastography. Type 1: predominantly not a blue lymph node, pathological diagnosis: reactive lymph node (**A**). Type 2: part blue, part not blue (green, yellow, red), pathological diagnosis: chronic granulomatous lymphadenitis (sarcoid) (**B**). Type 3: predominantly blue, malignant lymph node (**C**).

The number and size of lymph nodes to be sampled in thorax CT of all patients and SUV max values of lymph node/nodes to be sampled in patients with PET/CT were noted. After the target lymph node was detected with the EBUS elastography device (Hi-Vision Avius Elastography, Hitachi Medical Corporation, Japan), B-mode sonographic findings were recorded according to the localization, size, shape, border features, internal structure, presence of fatty hilar, and necrotic regions based on the study performed by Fujiwara et al. [13]. Lymph nodes were divided into stations as defined by the International Association for the Study of Lung Cancer (IASLC) [14]. Lymph nodes with round and noticeable borders, >1 cm, and hypoechoic internal structure were evaluated to be malignant. Those evaluations were made by an experienced bronchoscopist (EC). Then, real-time tissue elastography was obtained in the selected lymph node. The elastographic evaluation of Izumo et al. was taken into consideration during the classification of lymph nodes according to color distribution as follows: type 1, predominantly nonblue (green, yellow, and red) (Figure 1A); type 2, part blue, part nonblue (green, yellow and red) (Figure 1B); type 3, predominantly blue and shown with our examples (Figure 1C) [12]. After this evaluation, 1–4 samples were taken from each lymph node during EBUS (BD, EC).

### 2.3. Strain ratio (SR) calculation

**Figure 2:**
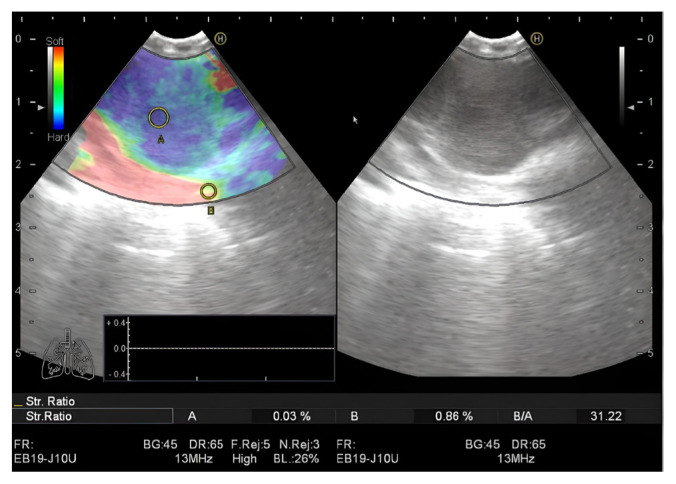
Calculation of the strain ratio (B/A) in the lymph node (A: pathological area; B: normal tissue area).

The area of interest for elastographic evaluation was selected by using a trackball and avoiding vascular structures. After an image without an artifact was obtained, the ‘freeze’ function was used and the most appropriate area of the lymph node was determined to measure the strain (area A). To determine SR as a reference, area B was determined from the normally visible area of the mediastinum surrounding the lymph node and SR (B/A) measurements were performed (Figure 2). During the EBUS elastography procedure, lymph nodes were evaluated by an experienced physician (EC, BD) and the target lymph nodes were sampled.

### 2.4. Statistical evaluation

Data analysis was performed using SPSS software (SPSS version 25; IBM Corporation, Armonk, NY, USA). Frequency (n) and percentage (%) values of demographic variables and mean and standard deviation values of quantitative variables were presented. The availability of the methods as a diagnostic test was tested by ROC analysis. It was concluded that the methods found to be significant as a result of the ROC analysis could be used as a diagnostic test. Kappa coefficient was used in the comparison between EBUS B mode and EBUS elastography findings with pathological diagnosis. In cases where the EBUS B mode is positive, the rate of giving a positive result of EBUS elastography is sensitivity; in cases where the EBUS B mode was negative, the rate of giving a negative result of EBUS elastography is specificity. In cases where EBUS elastography is positive, the EBUS B mode being positive is a positive predictive value (PPV); in cases where EBUS elastography is negative, the EBUS B mode being negative is a negative predictive value. The type I error rate was taken as 0.05 in the study and p-values less than 0.05 were considered to be significant.

## 3. Results

The average age of 131 patients included in the study was 55.86 ± 13 years, of which 76 (58%) were male and 55 (42%) were female. Among all patients, 45 (34.4%) were smokers, 42 (32.1%) were exsmokers and 44 (33.6%) were nonsmokers. The mean duration of smoking was 38.6 ± 28.9 packs/year. Eight (6.1%) patients had tuberculosis sequelae, 7 (5.3%) had extrapulmonary malignancies, 16 (12.2%) had diabetes, 38 (29%) had hypertension, 20 (15%) had chronic obstructive lung disease, 13 (9.9%) had ischemic heart disease, 6 (4.6%) had secondary diseases such as past cerebrovascular event. In 80 (61.5%) cases with hilar/mediastinal lymphadenopathy of unknown cause, diagnosis and staging were performed in 27 (20.6%) cases with a lung mass and mediastinal lymphadenopathy, and only staging was performed in 14 (10.6%) cases diagnosed with lung cancer, an investigation of recurrence was performed in 3 (2.3%) patients with a history of lung malignancy and a procedure was performed to investigate mediastinal metastasis in 7 (5.3%) patients with known extrapulmonary malignancies (renal cell, colon, endometrium, breast, larynx, and nasopharynx). Demographic characteristics and elastography indications of the patients are presented in Table 1. A total of 219 lymph nodes were sampled. The most common lymph node sampled among all stations was those from the subcarinal region.

When the mean size of the short axis was taken as 15.28 ± 7.4 mm and the short axis as ≥10 mm on thorax CT of the 219 lymph nodes evaluated for determining malignant LN, the sensitivity was 88%, specificity was 39.86%, PPV was 34.6%, NPV was 75% and the diagnostic accuracy was 35%. When the mean SUVmax value was taken as 15.21 ± 14.63 and FDG as ≥2.5 in PET/CT for determining malignant LN, the sensitivity was 98.67%, specificity was 11.90%, PPV was 70.4%, NPV was 87% and the diagnostic accuracy was 80.8%. The results of radiological imaging methods are presented in Table 2.

When sonographic findings were evaluated individually with EBUS B-mode, the diagnostic values of the lymph node with size >1 cm, round shape, well-defined and hypoechoic internal structure in the differentiation of malignant and benign lymph nodes were 48.4%, 58%, 51.6%, and 47%, respectively. When all EBUS B-mode sonographic findings and pathological results were compared, sensitivity was 65.33%, specificity was 63.19%, PPV was 48%, NPV was 77.8% and diagnostic value was 63%.

**Figure 3:**
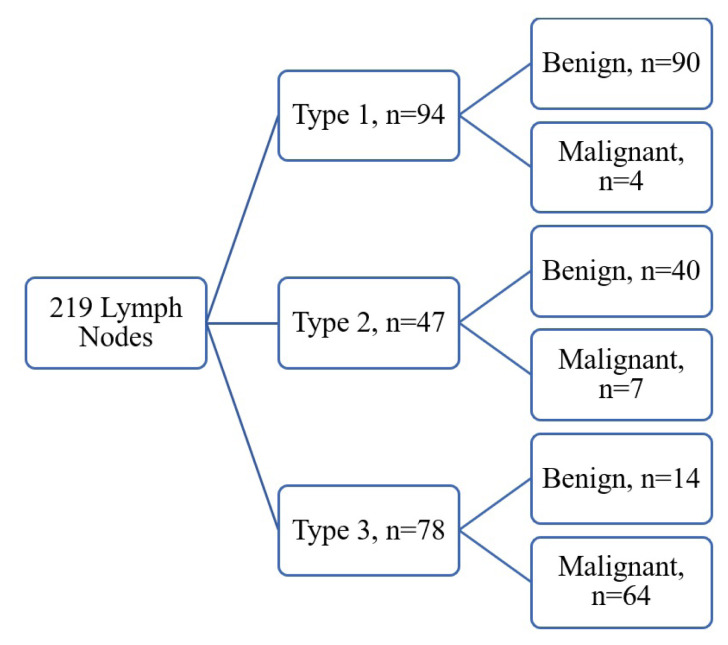
Distribution of lymph nodes according to Izumo classification and pathology results.

According to EBUS elastography findings, 94 (42.9%) lymph nodes were classified as type 1, 47 (21.5%) lymph nodes as type 2, and 78 (35.6%) lymph nodes as type 3 (Figure 3). Pathological diagnosis of 75 (34.2%) lymph nodes was malignant (17 small cell lung cancer, 17 unclassified, 12 squamous cell, 23 adenocarcinoma, 1 lymphoma, 2 mesothelioma, 1 epithelioid sarcoma, 2 prostate cancer metastasis) and 144 (65.8%) lymph nodes (44 granulomatous lymphadenites, 58 reactive lymph nodes, 41 anthracosis, 1 infective lymphadenitis) were detected to be benign. Patients who were pathologically diagnosed with benign or anthracotic lymph nodes were followed up. Mediastinoscopy was performed for 5 patients with clinical suspicion in the follow-up. The final pathology results of these patients were also reported as benign. The characteristics and pathology results of the lymph nodes sampled are presented in Table 3. When types 1 and 2 lymph nodes were evaluated to be benign and type 3 lymph nodes to be malignant, the sensitivity and specificity of elastography were calculated as 85.33% and 90.28%, respectively. When type 2 lymph nodes were ruled out and type 1 was evaluated to be benign and type 3 to be malignant, the sensitivity was 94.12%, specificity was 86.54%, PPV was 82.1%, NPV was 95.7% and the diagnostic value was 89.5%. The mean SR of the lymph nodes was 35.33 ± 46.10. SR of the malignant lymph nodes was statistically significantly higher compared to benign lymph nodes (71 ± 48.6, 16.75 ± 33.61, p = < 0.001). When the cutoff value was determined as 5.06 for SR, its sensitivity was calculated to be 94.67%, specificity to be 75.69%, PPV to be 96.5%, NPV to be 67%, diagnostic value to be 82.2% and AUC to be 0.867. The sensitivity, specificity, PPV, NPV, kappa, and AUC values of the methods we used to evaluate lymph nodes are presented in Table 4.

## 4. Discussion

We found that EBUS elastography alone has high diagnostic efficacy in the differentiation of malignant and benign lymph nodes. EBUS elastography had a higher diagnostic efficacy when compared to EBUS-B mode sonographic findings in the differentiation of malignant and benign lymph nodes. The coloring scale and SR were not superior to one another in distinguishing lymph nodes.

In the study performed by Izumo et al. in which they compared EBUS elastography and pathology results, group 1 lymph nodes were found to be 100% benign and group 3 lymph nodes were found to be 94.6% malignant [12]. In a similar study by Huang et al. in which they compared the elastography and pathological results of 78 lymph nodes, the diagnostic accuracy of elastography was found to be 96.3% benign in type 1 lymph nodes and 87.1% malignant in type 3 lymph nodes [15]. In another study, it was reported that 44.4% of the lymph nodes interpreted as type 1 were benign and 86.2% of the lymph nodes interpreted as type 3 were malignant [16]. In our study, type 1 lymph nodes were found to be 95.7% benign and type 3 lymph nodes to be 82.1% malignant. Our findings were consistent with the literature. Izumo and Huang showed that the diagnostic value of EBUS elastography increased significantly when they ruled out the lymph nodes determined as type 2 and evaluated type 1 as benign and type 3 as malignant. Similarly, Lin et al. found 90.6% sensitivity and 82.6% specificity by using the same criteria while Fournier et al. found 87% sensitivity and 68% specificity in a study in which they sampled a total of 217 lymph nodes [17,18]. When our study was evaluated together with other studies, it was observed that EBUS elastography increased the diagnostic efficacy in the prediction of malignant and benign lymph nodes with a sensitivity of 94.1% and a specificity of 86.5% when type 2 lymph nodes were ruled out (Table 5).

In previous studies, it was shown that SR was effective in differentiating benign and malignant lymph nodes. In the study performed by Ales et al., the mean SR for malignant lymph nodes was 18.96 ± 18.32 and 6.27 ± 7.30 for benign lymph nodes, and the area under the curve (AUC) was reported to be 0.870. When the cutoff value was taken as 8, the sensitivity was 88.24% and the specificity was 84.78% [19]. In the study performed by He et al., the mean SR was 87.69 ± 49.15 for malignant lymph nodes and 20.60 ± 17.14 for benign lymph nodes. AUC was reported to be 0.933. When the cutoff value was taken as 32.07, they obtained 88.1% sensitivity and 80.8% specificity [5]. In another single-center, prospective study, sensitivity was reported to be 86% and specificity to be 73% when the cutoff value was taken as 5.3. AUC value was reported to be 0.850 [20]. In the study performed by Çağlayan et al. on 93 patients diagnosed with malignancy, the sensitivity for malignancy was 75% and the specificity was 65% with an SR cutoff value of ≥ 2.47 [21]. In our study, the sensitivity was 94.67%, the specificity was 75.69% and the AUC was 0.867 when the cutoff value was taken as 5.06 for SR. Similar to previous studies, SR was found to be effective in distinguishing benign and malignant lymph nodes but no superiority was found over the color scale. However, in cases where the color scale alone is not sufficient to predict the diagnosis, it was considered that SR could provide control to the operator and could be used as a support for the diagnosis since they have similar sensitivities.

According to the EBUS lymph node sonographic features defined in the study performed by Fujiwara et al., it was reported that 42.9% of the lymph nodes with at least one of the four categories were pathologically malignant and 96% of the lymph nodes that did not contain all four categories were pathologically benign [13]. However, it was also reported that the diagnostic efficacy ranged between 40% and 80% in studies conducted with the classification of each B-mode finding [1–3]. Similar to these studies, the diagnostic efficacy of classification by each B-mode finding was found to be 47%–60.3% and deemed to be compatible with the literature in our study.

He et al. compared the sensitivity and specificity of EBUS elastography with conventional B-mode imaging in the differentiation of malignancies. They reported that the elastography grading score had a higher sensitivity and specificity than conventional B-mode criteria in the diagnosis of malignant lung lesions [5, 22]. When each B mode was evaluated individually, the diagnostic efficacy was 47%–60.3% and when all sonographic features were evaluated together, the diagnostic efficiency was 63% in our study while the diagnostic efficacy of EBUS elastography was 89.5% which was observed to be superior.

The limitations of our study include the fact that the study has been conducted in a single center and the pathological results of EBUS-TBNA have been taken as a reference for the definitive diagnosis of lymph nodes. Another limitation is that since the SR is determined from frozen EBUS images, selection bias may occur especially during the selection of the reference area in SR evaluation and the static image selection.

In conclusion, it was observed that EBUS elastography could be used alone with high efficacy and high diagnostic yield in the differentiation of malignant and benign lymph nodes. It had superior diagnostic efficacy compared to EBUS-B mode sonographic findings. Although the coloring scale and SR are not superior to one another in distinguishing lymph nodes, it should be noted that they can be used as supporting procedures for each other in cases where they cannot predict the diagnosis by themselves. It was also considered that it would be beneficial if the use of EBUS elastography were common. In line with our findings, future studies including patients with histopathological diagnoses having larger sample sizes should be planned. In addition, it would be better to demonstrate the discriminative ability of elastography in the diagnosis of antracotic and granulomatous diseases.

## Figures and Tables

**Table 1:** Demographic characteristics and elastography indications of the patients.

**Age (mean, SD)**	55.86 ± 13
**Sex (n, %)**	
Female	55 (42)
Male	76 (58)
**Smoking history (n, %)**	
Smoker	45 (34.4)
Nonsmoker	44 (33.6)
Exsmoker	42 (32.1)
Pack/year (mean ± SD)	38.6 ± 28.9
**Comorbidities (n, %)**	
HT	38 (29)
COPD	20 (15)
DM	16 (12.2)
IHD	13 (9.9)
Extrapulmonary malignancy	7 (5.3)
Sequelae tuberculosis	8 (6.1)
Past CVE	6 (4.6)
**Indications (n, %)**	
Diagnosis	80 (61.5)
Diagnosis and staging	27 (20.6)
Staging	14 (10.6)
Recurrence or progression of disease	3 (2.29)
Metastasis of extrapulmonary malignancy	7 (5.34)

COPD: chronic obstructive pulmonary disease, CRF: chronic renal failure, CVE: cerebrovascular event, DM: diabetes mellitus, HT: hypertension, IHD: ischemic heart disease, SD: standard deviation.

**Table 2:** Sensitivity, specificity, NPV, and PPV of parameters in thorax CT and PET/CT in the prediction of malignant and benign hilar and mediastinal lymph nodes.

		Mean SD (mm/SUVmax)	Sensitivity	Specificity	PPV	NPV
Thoracic CT	Short axis ≥ 10 mm	15.28 ± 7.40	88	39.9	34.6	75
PET/CT	SUVmax ≥ 2.5	15.21 ± 14.63	98.7	11.9	50	98.6

CT: computed tomography, NPV: negative predictive value, PET-CT: positron emission tomography-computed tomography, PPV: positive predictive value, SUVmax: standardized uptake value maximum.

**Table 3:** Characteristics of hilar and mediastinal lymph nodes.

**Number (n)**	219
**Stations (n, %)**	
2R (right upper paratracheal)	4 (1.8)
4R (right lower paratracheal)	55 (25.1)
4L (left lower paratracheal)	10 (4.6)
7 (subcarinal)	90 (41.1)
10R (right hilar)	5 (2.3)
10L (left hilar)	4 (1.8)
11R (right interlobar)	29 (22.1)
11L (left interlobar)	20 (9.1)
Mass	2 (0.9)
**Elastography**	
Type 1	94 (42.9)
Type 2	47 (21.5)
Type 3	78 (35.6)
**SR (mean** ± **SD)**	35.33 ± 46.10
**Pathological diagnoses**	
**Malignant (n, %)**	
Small cell lung cancer	17 (22.6)
Nonsmall cell lung cancer	52 (69.2)
Squamous cell lung cancer	12 (16)
Adenocarcinoma	23 (30.6)
Unclassified	17 (22.6)
Mesothelioma	2 (2.66)
Lymphoma	1 (1.33)
Prostate metastasis	2 (2.66)
Epithelioid sarcoma	1 (1.33)
**Benign (n, %)**	
Chronic granulomatous lymphadenitis	44 (30.56)
Tuberculosis	6 (4.17)
Sarcoidosis	38 (26.39)
Anthracosis	41 (28.4)
Reactive	58 (40.2)
Nonspecific lymphadenitis	1 (0.69)

SD: standard deviation, SR: strain ratio.

**Table 4:** Sensitivity, specificity, NPV, PPV, diagnostic and p values of elastography (ignoring type 2), SR, and B-mode sonographic findings in predicting whether hilar and mediastinal lymph nodes are malignant or benign.

Classification			Sensitivity	Specificity	PPV	NPV	Diagnostic value	AUC (95%CI)/Kappa	p
EBUS	Elastography		94.1	86.5	82.1	95.7	89.5	0.787^K^	< 0.001
	SR cutoff > 5.06		94.7	75.7	67	96.5	82.2	0.867 (0.814 −0.909)^A^	< 0.001
	B-mode	Shape	86.7	43.1	44.2	86.1	58	0.242^K^	< 0.001
		Border	92.0	30.6	40.8	88	51.6	0.173^K^	< 0.001
		Echogenicity	94.7	22.2	38.8	88.9	47	0.126^K^	< 0.001
		Size > 1 cm	81.3	31.3	38.1	76.3	48.4	0.099^K^	0.314
		Presence of fatty hilus	0	91.7	0	63.8	60.3	−0.104^K^	0.010
		Presence of necrosis	16.2	89.6	44.4	67.5	64.7	0.069^K^	0.272
		Shape + Border + Echogenicity + Size	65.3	63.2	48	77.8	63	0.263^K^	< 0.001

A AUC, AUC: area under the curve,

K kappa,

NPV: negative predictive value, PPV: positive predictive value, SR: strain ratio.

**Table 5:** Type 1 (benign)/Type 3 (malign) and sensitivity, specificity of elastography (ignoring type 2) in studies.

Studies	Type 1 (benign)	Type 3 (malign)
Izumo et al.	100%	94.6%
Huang et al.	96.3%	87.1%
Gompelmann et al.	44.4%	86.2%
Our study	95.7%	82.1%
	**Sensitivity**	**Specificity**
Lin et al.	90.6%	82.6%
Fournier et al.	87%	68%
Our study	94.1%	86.5%
